# Clinical application and drug resistance mechanism of gemcitabine

**DOI:** 10.3389/fcell.2025.1702720

**Published:** 2025-11-10

**Authors:** Xuanrui Zhang, Bing Qi, Jing Chen

**Affiliations:** College of Life Sciences, North China University of Science and Technology, Tangshan, Hebei, China

**Keywords:** gemcitabine, resistance mechanism, tumor microenvironment, combination therapy, precision medicine

## Abstract

Gemcitabine, as a nucleoside analog, exerts a broad-spectrum antitumor effect by interfering with DNA synthesis, but its clinical application is limited by drug resistance. The drug resistance mechanism involves metabolic abnormalities (such as downregulation of deoxycytidine kinase (dCK), nucleoside transporter hENT1 deficiency), enhanced DNA repair (overexpression of ribonucleotide reductase ribonucleotide reductase catalytic subunit M1 (RRM1)/ribonucleotide reductase catalytic subunit M2 (RRM2), and tumor microenvironment remodeling (such as secretion of immunosuppressive factors by CAFs). This article systematically reviews the drug resistance mechanism of gemcitabine and explores the breakthrough direction of new drug delivery systems (liposomes, albumin nanoparticles) and combination therapy strategies (targeted drugs, immune checkpoint inhibitors). In addition, cutting-edge technologies such as single-cell sequencing and artificial intelligence drug sensitivity prediction provide a new paradigm for precision treatment. In the future, it is necessary to build a “prevention-monitoring-intervention” full-chain management system through dynamic monitoring of multi-omics biomarkers (such as circulating tumor DNA tracking RRM2 amplification) and coordinated intervention of traditional Chinese and Western medicine (such as curcumin reversing drug resistance).

## 1 Introduction

### 1.1 Introduction to gemcitabine

**FIGURE 1:**
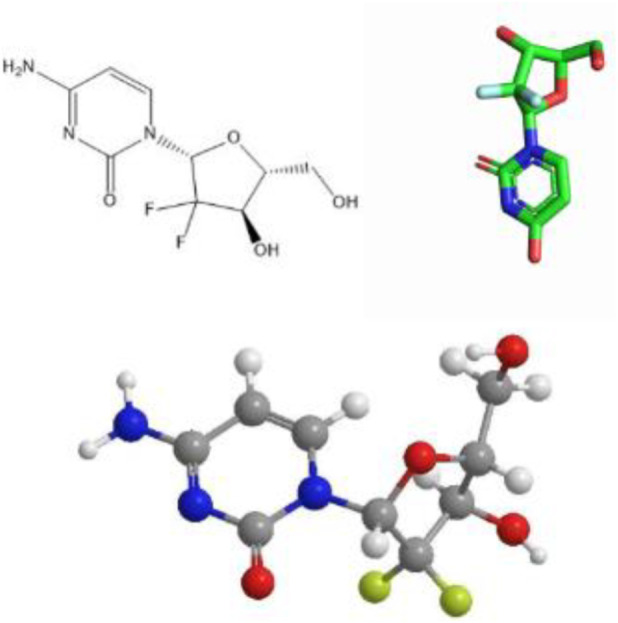
Schematic diagram of the chemical structure of gemcitabine.

Gemcitabine (chemical formula: C_9_H_11_F_2_N_3_O_4_·HCl) is a cytosine nucleoside analog that exerts its antitumor effect by interfering with nucleotide metabolic pathways. Its chemical structure is shown in Figure 1. The drug was first approved for clinical use in the UK in 1995, and was subsequently approved by the US Food and Drug Administration (FDA) for the treatment of a variety of solid tumors: pancreatic cancer in 1996 (Li W. et al., 2020), expanded to non-small cell lung cancer in 1998, and further approved for metastatic breast cancer in 2004 (Principe et al., 2021). As a core drug in first-line/second-line chemotherapy regimens, gemcitabine has shown broad-spectrum antitumor activity in the treatment of solid tumors such as non-small cell lung cancer, pancreatic cancer, and ovarian cancer.

Notably, gemcitabine exerts cytotoxic effects primarily by inhibiting DNA synthesis and also demonstrates significant antiviral activity. Studies have shown that it can inhibit the replication of hepatitis E virus by upregulating the type I interferon signaling pathway (Li Y. et al., 2020). This multi-target mechanism of action makes it a unique chemotherapy drug with both antitumor and anti-viral potential.

### 1.2 Clinical application status and history of gemcitabine

As early as in the Burris trial in 1997, gemcitabine was compared with 5-fluorouracil (5-FU) monotherapy. The overall survival (OS) of gemcitabine group was extended to 5.65 months (vs. 4.41 months), and the quality of life (QoL) was improved, establishing it as the cornerstone of first-line treatment for pancreatic ductal adenocarcinoma (PDAC) (Madoff et al., 2022; Zhang et al., 2019).

Between the 2000s and 2010s, as gemcitabine showed good antitumor activity and tolerability, it began to be extended to other solid tumors: In 2002, the FDA approved the combination of gemcitabine and cisplatin for non-small cell lung cancer (NSCLC), and compared with cisplatin alone, the OS was significantly prolonged, becoming one of the first-line standard regimens for NSCLC (Gou et al., 2024). In 2004, the FDA approved gemcitabine + paclitaxel for metastatic breast cancer (MBC), replacing paclitaxel alone and prolonging survival (Yang Y. et al., 2023). In the late 2000s, gemcitabine + cisplatin became the standard chemotherapy regimen for bladder cancer, compared with the traditional MVAC regimen (methotrexate + vinblastine + doxorubicin + cisplatin), it has lower toxicity and similar efficacy (Xia et al., 2022).

Since the 2010s, with the increasing recognition of gemcitabine resistance, researchers have explored various combination therapies. Classic combination chemotherapy regimen: The FOLFIRINOX regimen (oxaliplatin + irinotecan + 5-FU/leucovorin) significantly improved outcomes versus gemcitabine, with median OS of 11.1 months vs. 6.8 months, PFS of 6.4 vs. 3.3 months, and ORR of 31.6% vs. 9.4% (HR 0.57, *p* < 0.001), albeit with higher grade 3–4 toxicity (neutropenia 45.7%) (Huffman et al., 2022; Conroy et al., 2011). Similarly, the MPACT trial (NCT00844649) showed that gemcitabine + nab-paclitaxel (AG regimen) extended median OS to 8.5 months vs. 6.7 months with gemcitabine, with ORR of 23% vs. 7%, and manageable toxicity (grade ≥3 neutropenia 38%) (Cui et al., 2020; Von Hoff et al., 2013). In 2019, a study on the efficacy and economy of FOLFIRINOX regimen in advanced pancreatic cancer showed that its total survival time (OS) benefit varied with disease stage, and the median OS of metastatic patients was 11.1 months, significantly better than gemcitabine alone; The median OS of locally advanced patients was 15.7 months, with no statistically significant difference compared to gemcitabine. The median OS of critically resectable patients reached 37 months, accompanied by a higher surgical conversion rate (Giuliani and Bonetti, 2019). The latest high-level evidence shows that the dominance of FOLFIRINOX in advanced pancreatic cancer is being challenged and tends to be refined. In Asian metastatic pancreatic cancer population, JCOG1611 Phase III study showed that the median OS of AG protocol (albumin paclitaxel + gemcitabine) was 17.1 months, significantly better than that of mFOLFIRINOX for 14.0 months (Ohba et al., 2025). However, in locally advanced pancreatic cancer, FOLFIRINOX can still significantly improve progression free survival (PFS). For critically resectable patients, it still demonstrates irreplaceable value in providing excellent surgical conversion rates and long-term survival (such as a median OS of 37 months in Indian studies) (Chiorean et al., 2019). The current treatment strategy has shifted from “single protocol priority” to individualized selection based on disease staging, regional population differences (Asian and non Asian), and biomarkers (such as BRCA status).

In terms of targeted therapy, in addition to PARP inhibitors (Olaparib) for pancreatic cancer patients with BRCA mutations, gemcitabine can significantly prolong progression-free survival (PFS) when used in combination with it, CXCR4 inhibitors can improve the tumor microenvironment and increase gemcitabine sensitivity, and Hedgehog signaling pathway inhibitors: such as Vismodegib, can remove the matrix barrier and improve the delivery efficiency of gemcitabine (Wang et al., 2023).

In terms of immunotherapy, the use of PD-1(Programmed Cell Death Protein 1) inhibitors (such as Keytruda) alone has poor effects on PDAC, but combination with gemcitabine can enhance immune activation (Zhang et al., 2022). For example, gemcitabine + Durvalumab (Programmed Death-Ligand 1 inhibitor) + Tremelimumab (Cytotoxic T-Lymphocyte-Associated Protein 4 inhibitor) has shown good efficacy in some patients (Powles et al., 2020).

Gemcitabine hydrochloride is generally administered by intravenous injection in clinical practice. It is used as the first-line drug for locally advanced (stage III) and metastatic (stage IV) non-small cell lung cancer, and as the second-line drug for patients with advanced pancreatic cancer (Perez et al., 2013). Gemcitabine can be rapidly distributed to various tissues in the body after intravenous injection, has a long half-life, and is less toxic to patients. It has long been widely used in clinical practice.

At present, the clinical application of gemcitabine is generally in the form of two-drug combination, three-drug combination or related to immunochemotherapy, among which three-drug combination is used. Under the targeted induction of the vector, it has highly selective inhibitory effect on bladder cancer cells (Petrosyan et al., 2022). Gemcitabine remains a cornerstone of both clinical practice and laboratory research, and it is expected to maintain its significant role in future oncology therapeutics.

### 1.3 The significance of studying the mechanism of action and drug resistance of gemcitabine

Gemcitabine has a low oral bioavailability of about 10% and is, thus, administered via intravenous infusion, typically once per week at a dose of 1,000–1,250 mg/m^2^ (Thompson et al., 2020), actively exploring its mechanism of action and improving it is the focus of current research. We also believe that studying the intracellular drug delivery mechanism is of great help in addressing drug resistance. For example, changes in the expression of proteins that transport drugs into cells (SLC29A1, SLC28A1, and SLC28A3) seem to lead to changes in its efficacy (Alvarellos et al., 2014; Mini et al., 2006). Similarly, the expression of proteins that cause its inactivation (such as deoxycytidine deaminase), and the expression of other intracellular target genes can lead to changes in response to the drug. The study and elucidation of the mechanism will provide new ideas and methods for treatment.

Although gemcitabine has good antitumor effects, drug resistance has always been a difficult problem in clinical treatment. Drug resistance leads to treatment failure in more than 60% of advanced patients, and the median survival of key indications (such as pancreatic cancer) is still less than 1 year (Garcia-Carbonero et al., 2022). Although we have adopted a variety of methods to reduce the impact of drug resistance, such as combination therapy, drug carriers, and immune methods, the problem of drug resistance has not been cured. Consequently, research into drug resistance mechanisms has critical implications for drug development and the optimization of chemotherapy regimens.

### 1.4 Literature search strategy

A comprehensive literature search was conducted to identify relevant publications up to September 2025 from the electronic databases PubMed, Web of Science, and Google Scholar. The search terms included a combination of the following keywords and their variants: “gemcitabine,” “resistance,” “mechanism,” “tumor microenvironment,” “RRM2,” “RRM1,” “human equilibrative nucleoside transporter 1 (hENT1),” “dCK,” “nanoparticle,” “combination therapy,” “immunotherapy,” along with specific cancer types such as “pancreatic cancer,” “non-small cell lung cancer,” and “bladder cancer.” The search was limited to articles published in English. The inclusion criteria focused on original research articles, high-quality reviews, and key clinical trials that provided insights into the mechanisms of action, resistance, and novel therapeutic strategies of gemcitabine. We excluded studies that were considered to have poor methodological quality and conference abstracts without full texts.

## 2 Progress in clinical application of gemcitabine and related drug resistance mechanisms

### 2.1 Clinical indications in different cancer types

#### 2.1.1 Pancreatic cancer

PDAC is an aggressive malignancy characterized by poor prognosis, patients with PDAC have a 5-year survival rate of 10% (Bugazia et al., 2024). The characteristic pathological changes of this disease are a highly fibrotic tumor microenvironment (TME), in which cancer-associated fibroblasts (CAFs) form a dense physical barrier by secreting extracellular matrix (ECM) components such as hyaluronic acid and type IV collagen, significantly reducing the penetration efficiency of chemotherapeutic drugs (Basar et al., 2024). Moreover, PDAC is typically hypovascular, which further constrains perfusion-dependent delivery of systemic agents (Obaid et al., 2022; Annese et al., 2019).

Since its approval for PDAC in 1997, single-agent gemcitabine has remained a cornerstone of systemic therapy; historically, gemcitabine monotherapy yielded a median overall survival (OS) of approximately 5.65 months, with a 1-year survival rate of 18% (Hassan et al., 2023; Burris et al., 1997). With the development of combined therapy strategies, the FOLFIRINOX regimen (5-fluorouracil + oxaliplatin + irinotecan) and the AG regimen (gemcitabine + albumin-bound paclitaxel) have become new standards for first-line treatment. In FOLFIRINOX regimen, the median overall survival was 11.1 months in the FOLFIRINOX group as compared with 6.8 months in the gemcitabine group (hazard ratio for death, 0.57; 95% confidence interval [CI], 0.45 to 0.73; P < 0.001). Median progression-free survival was 6.4 months in the FOLFIRINOX group and 3.3 months in the gemcitabine group (hazard ratio for disease progression, 0.47; 95% CI, 0.37 to 0.59; P < 0.001). The objective response rate was 31.6% in the FOLFIRINOX group versus 9.4% in the gemcitabine group (P < 0.001). More adverse events were noted in the FOLFIRINOX group; 5.4% of patients in this group had febrile neutropenia. At 6 months, 31% of the patients in the FOLFIRINOX group had a definitive degradation of the quality of life versus 66% in the gemcitabine group (hazard ratio, 0.47; 95% CI, 0.30 to 0.70; P < 0.001) (Ye et al., 2024; Möhr et al., 2023). In the phase III MPACT trial, gemcitabine plus nab-paclitaxel (AG) significantly improved outcomes compared with gemcitabine alone, with a median OS of 8.5 vs. 6.7 months (hazard ratio 0.72, P < 0.001), median PFS 5.5 vs. 3.7 months, and objective response rate 23% vs 7%. The main grade ≥3 toxicities were neutropenia 38%, fatigue 17%, and peripheral neuropathy 17% (Von Hoff et al., 2013). A retrospective study of patients with locally advanced and metastatic pancreatic cancer treated with nab-paclitaxel (125 mg/m^2^) plus gemcitabine (1,000 mg/m^2^) on days 1 and 8 every 21-day at West China Hospital and Shang Jin Hospital of Sichuan University from March 2018 to December 2021 were reviewed retrospectively (Retrospective). Clinical characteristics of patients were collected. The progression-free survival, overall survival, objective response rate, disease control rate, and toxicity were evaluated. A total of 113 patients who received the modified regimen of 21-day nab-P/Gem chemotherapy were included. The median overall survival was 9.3 months and the median progression-free survival was 4.4 months. The objective response rate and disease control rate were 18.6% and 56.7%, respectively. The median relative dose intensity for this modified regimen was 65%. The adverse events were mild to moderate, and the most common grade 3 or 4 treatment-related adverse events were neutropenia (21%) and leukopenia (16%) (Chang et al., 2022).

Gemcitabine resistance is common in PDAC, involving multiple biological mechanisms: The first is TME -mediated drug delivery barriers: CAFs-driven ECM deposition and low angiogenesis significantly reduce gemcitabine concentrations in the tumor core area (Preclinical) (Huo et al., 2022). Although the ESPAC-3 clinical trial confirmed that high expression of human equilibrative nucleoside transporter 1 (hENT1) was positively correlated with drug sensitivity (HR = 0.55, p < 0.01) (Tamura et al., 2015; O et al., 2024), the ECM barrier effect caused by TME heterogeneity may weaken its predictive value (Azizi et al., 2023).

Another mechanism of drug resistance is related to cancer stem cells (CSCs): ALDH1A1-positive CSCs escape drug killing through multiple pathways: 1. high expression of efflux pump proteins such as ABCG2. 2. activation of BRCA mediated DNA damage repair pathways and 3. interaction with TME through Notch/Hedgehog signaling to maintain stemness characteristics (Gu X. et al., 2024).

There are also abnormal epigenetic regulation: overexpression of RRM2 antagonizes drug effects by increasing the dNTP pool, while epigenetic silencing of deoxycytidine kinase (dCK) (such as promoter hypermethylation) leads to drug activation disorders (Shen et al., 2022).

In view of the above-mentioned drug resistance mechanisms, the following intervention strategies are currently adopted:

TME targeted therapy: Use PEGylated hyaluronidase (PEGPH20) to degrade ECM components (NCT02715804) (Liu L. et al., 2022) or FAK inhibitors to improve immune cell infiltration (Li et al., 2022). CSC elimination strategy: Combined with ALDH inhibitor disulfiram (Bamodu et al., 2024), Hedgehog pathway inhibitor Vismodegib (Basar et al., 2024), or PD-1 inhibitors to reshape the immune microenvironment. Novel delivery system: Albumin nanoparticles bypass hENT1-dependent uptake through gp60 receptor -mediated transmembrane transport (Yu et al., 2015). Precision medicine application: Dynamic monitoring of RRM2 gene copy number variation based on ctDNA (Papaccio et al., 2022), and the BRCA mutation stratified treatment model established by the POLO trial (NCT02184195) (Keane et al., 2023).

The development of oral nano formulations of gemcitabine faces multiple challenges, including strong hydrophilicity, poor gastrointestinal stability, and low oral bioavailability due to the intestinal epithelial barrier. In addition, the nano formulations have difficulties in controlling critical quality attributes (CQA) between batches, low drug loading, and insufficient process scaling stability in GMP (Good Manufacturing Practice) production. In recent years, researchers have addressed these challenges through various innovative strategies, such as constructing self-assembled prodrug nanosystems (such as gemcitabine Fmoc amino acid conjugates GWC/GPC/GLC) to improve loading efficiency and metabolic stability without relying on external carriers (Bano et al., 2025). Using precise nanotechnology (such as DNA solid-phase synthesis to prepare structurally clear poly to achieve chemical component uniformity and controlled drug release (Chen K. et al., 2022). Adopting active targeting strategies (such as modifying iRGD peptides or glycocholic acid GCA) to enhance intestinal absorption and tumor targeting ability by activating specific transporters (such as ASBT, OCTN2) (Wang et al., 2020). And by utilizing the Design of Experiments (DoE) method, the prescription process is systematically optimized to enhance the stability and reproducibility of nano formulations. However, its clinical translation still needs to further address key issues such as the safety of carrier materials, the reliability of large-scale production, and comprehensive quality evaluation that meets regulatory requirements.

In the field of neoadjuvant therapy, the PRODIGE 24 phase III clinical trial (NCT01526135) confirmed that the neoadjuvant FOLFIRINOX regimen significantly prolonged disease-free survival compared with postoperative gemcitabine alone (21.6 vs. 12.8 months) (Casolino and Biankin, 2023), but its hematological toxicity limits the selection of patients with good physical condition. Future research directions should acknowledge that, to date, immune checkpoint inhibitors have not demonstrated a solid randomized benefit in unselected PDAC populations.

#### 2.1.2 Non-small cell lung cancer

In non-small cell lung cancer, gemcitabine is often used in combination with platinum drugs such as cisplatin or carboplatin, especially for patients with unresectable or advanced disease. Several clinical studies have shown that this combination chemotherapy regimen can improve tumor response rates and prolong progression - free survival in some patients (Boonsong et al., 2022).

As a commonly used drug for chemotherapy of NSCLC, gemcitabine exerts its antitumor effect by interfering with DNA synthesis, but its efficacy is often limited by tumor microenvironment remodeling and drug resistance mechanisms. On the one hand, epithelial-mesenchymal transition (EMT) promotes tumor cells to acquire invasiveness and drug resistance under the activation of the TGF-β(Transforming Growth Factor-beta) pathway (such as Smad2/3 phosphorylation inducing transcription factors such as Snail and ZEB1) (Si et al., 2022). EMT leads to cell cycle arrest (reducing drug sensitivity in the S phase) and increased drug efflux (upregulation of ABC transporters) (Zhang et al., 2021), while immunosuppression (inhibiting T/NK cells and promoting Treg differentiation) further weaken the efficacy (Jiang et al., 2020). On the other hand, there are cross-mechanisms between gemcitabine and platinum resistance, especially high ERCC1 expression leads to platinum resistance by enhancing DNA repair ability (such as repairing platinum-induced DNA cross-linking damage) (Böttger et al., 2024). Clinical data show that patients with high ERCC1 expression have a poor response to gemcitabine + platinum regimen (Das et al., 2023). In view of these mechanisms, strategies such as combining TGF-β inhibitors (such as Galunisertib), ERCC1 inhibitors or immune checkpoint blockade (PD-1/PD-L1 inhibitors) are expected to reverse resistance, but further research is needed to verify their synergistic effects and clinical application potential.

#### 2.1.3 Bladder cancer

Gemcitabine is still the cornerstone drug for bladder cancer chemotherapy, especially in patients with cisplatin intolerance (Ren et al., 2022). Its combination with immunotherapy and targeted drugs is the key direction for optimizing efficacy in the future.

As the main alternative for cisplatin -intolerant bladder cancer patients (such as GCb regimen), the efficacy of gemcitabine is limited by compensatory resistance (Zou et al., 2024). Resistance mechanisms include abnormal drug metabolism (such as downregulation of deoxycytidine kinase, nucleoside transporter defects), activation of DNA repair pathways (nucleotide excision repair NER/homologous recombination repair HRR), enhanced pro-survival signals (PI3K/AKT/mTOR), and tumor microenvironment remodeling (such as CAFs secreting immunosuppressive factors) (Chern and Tai, 2020). In addition, epithelial-mesenchymal transition (EMT) may further drive invasiveness and stem cell characteristics. To overcome resistance, clinical exploration of combined targeted therapy (such as PARP/PI3K inhibitors) or immunomodulation (such as combined use of PD-L1 inhibitors) is being conducted to enhance efficacy by intervening in key pathways or reversing the immunosuppressive microenvironment.

Platinum based drugs temporarily upregulate PD-L1 expression in tumor cells by activating DNA damage response pathways (such as ATM/ATR) and their downstream STAT3/NF - κ B signaling (Sato et al., 2020; Shin et al., 2019). However, the spatiotemporal heterogeneity caused by chemotherapy (such as differences in the distribution of immune cells within tumors) and clonal selection under treatment pressure (such as enrichment of low immunogenic subclones) (McGranahan et al., 2016) may weaken its synergistic effect with immune checkpoint inhibitors. Mechanistically, platinum based chemotherapy can enhance the immunosuppressive microenvironment, including promoting the expansion of Treg cells and myeloid derived suppressor cells (MDSCs) (Bie et al., 2023), inducing lymphocyte depletion (Ge et al., 2023), and suppressing MHC-I expression through epigenetic silencing (such as DNA methylation) (Song et al., 2024). The following table is a summary of the above content. We use Table 1 for a comparison so that we can see the differences more intuitively.

**TABLE 1:** Comparison of drug resistance mechanisms and coping strategies in different cancer types (pancreatic cancer, non-small cell lung cancer, bladder cancer).

Cancer type	Key resistance mechanisms	Coping strategy
Pancreatic cancer	Loss of hENT1 expression	Nano delivery system (albumin nanoparticles)
	Overexpression of RRM2	CD40 agonist combined with immunotherapy
	CAFs barrier	PEGPH20 degrades ECM
Non-small cell lung cancer	EMT mediated upregulation of ABC transporter protein	TGF - β inhibitor (Galunisterib)
	High expression of ERCC1	ERCC1 inhibitor combined with platinum based drugs
Bladder cancer	Activation of DNA repair pathway (HRR/NER)	PARP inhibitor (Olaparib)
	PI3K/AKT signal enhancement	PD-L1 inhibitor combined with chemotherapy

#### 2.1.4 EMT driven drug-resistant molecular network

Based on the current clinical situation, EMT is still one of the important reasons for clinical treatment problems, and further discussion is needed here. EMT reshapes the phenotype of tumor cells through multiple signaling pathways, and its drug resistance mechanism involves the following core links:

TGF-β/Smad pathway: TGF-β ligands (such as TGF-β1) bind to the cell membrane type II receptor (TβRII), recruit and phosphorylate the type I receptor (TβRⅠ), and activate the Smad2/3 complex. Phosphorylated Smad2/3 forms a heterotrimer with Smad4, translocates to the nucleus, directly binds to the promoters of the Snail and ZEB1 genes, and induces their transcription (Lamouille et al., 2014).

Wnt/β-catenin pathway: Wnt ligands (such as Wnt3a) inhibit the β-catenin degradation complex (APC/Axin/GSK3β), causing β-catenin to accumulate in the cytoplasm and enter the nucleus, bind to TCF/LEF transcription factors, upregulate the expression of Vimentin and N-cadherin, and inhibit E-cadherin (Nieto et al., 2016).

Cross-regulatory nodes: Snail induces chromatin compaction and transcriptional silencing by recruiting histone deacetylases (HDAC1/2) to the E-cadherin promoter region. ZEB1 forms a complex with YAP/TAZ to synergistically activate ABC transporter (such as ABCG2) expression and enhance drug efflux.

EMT interacts with the microenvironment: EMT-transformed cells activate tumor-associated fibroblasts (CAFs) by secreting factors such as TGF-β and IL-6, which in turn secrete HGF and FGF, maintaining the EMT phenotype through c-Met/FGFR signals, forming a positive feedback loop of drug resistance (Derynck et al., 2021).

### 2.2 Exploration of the application of comprehensive therapy

#### 2.2.1 Combined use with other chemotherapy drugs

Gemcitabine is often used in combination with cisplatin or carboplatin for the treatment of solid tumors such as NSCLC. Randomized phase III trials (NCT00087711) have shown that this regimen significantly enhances chemotherapy efficacy, improves objective response rate (ORR 32% vs. 18%), and prolongs progression free survival (median PFS 8.2 months) (Scagli et al., 2008). Especially in pancreatic cancer, the combination of gemcitabine and albumin bound paclitaxel has become one of the first-line standard protocols. The 10-year follow-up data of the MPACT trial showed that the median overall survival (OS) of the combination therapy group was 8.7 months, and the patient’s quality of life score was significantly better than that of the gemcitabine monotherapy group (Elsayed and Abdelrahim, 2021). In addition, preclinical studies have shown that the combination of gemcitabine and PARP inhibitors (such as olaparib) can reverse platinum resistance and enhance efficacy by targeting homologous recombination deficient (HRD) tumors (Liu G. et al., 2022). Other combination strategies, such as the use of docetaxel or fluorouracil, have also shown potential in refractory tumors (Jeong and Kim, 2020; Zhong et al., 2017).

#### 2.2.2 Combination therapy with targeted drugs

In recent years, the combination therapy of gemcitabine and various targeted drugs has been continuously explored. For example, in pancreatic cancer and some lung cancer, gemcitabine combined with the EGFR inhibitor erlotinib has been proved by the phase III clinical trial to significantly extend the median total survival period of patients (9.6 vs. 6.8 months) (Moore et al., 2007). However, due to its limited clinical impact and increased toxicity, this combination is not widely adopted in practice. Mechanistically, erlotinib enhances gemcitabine induced tumor cell apoptosis by inhibiting the EGFR signaling pathway and downregulating BRCA1 mediated DNA repair ability (Moore et al., 2007). In addition, for tumors with DNA repair defects, PARP inhibitors (such as Nilapalli) combined with gemcitabine can produce a synthetic lethal effect. Phase II test showed that the objective response rate of patients with BRCA mutation pancreatic cancer increased to 48% (O'Reilly et al., 2020). In anti angiogenic therapy, drugs such as bevacizumab reduce tumor angiogenesis by inhibiting the VEGF signaling pathway, and when combined with gemcitabine, can significantly prolong the progression free survival of patients with advanced biliary tract cancer (8.5 vs. 5.7 months) (Cheng et al., 2021). These joint strategies provide new directions for overcoming drug resistance and improving efficacy.

#### 2.2.3 Combination therapy with immunotherapy

Studies have shown that gemcitabine can induce immunogenic cell death, release tumor-associated antigens and activate antitumor immune (Oei and Schad, 2023). In addition, gemcitabine can reduce the number of immunosuppressive cells (such as myeloid suppressor cells) in the tumor microenvironment to a certain extent, thereby improving the local immune status and converting “cold” tumors into “hot” tumors (referring to tumors with less immune cell infiltration, while “hot tumors” contain abundant immune cells (Chen and Mellman, 2013), creating conditions for the application of immunotherapy drugs (such as PD-1/PD-L1 inhibitors). Notably, this effect has been confirmed in models of lung cancer and bladder cancer. However, in pancreatic cancer, this immunomodulatory effect is often insufficient to overcome the profoundly immunosuppressive TME, and the conversion to an immunologically ‘hot’ state is less common.

In terms of clinical treatment, the combination of existing immune checkpoint inhibitors (such as PD-1/PD-L1 antibody) and gemcitabine chemotherapy scheme has not shown a stable and significant therapeutic advantage in unselected patients with pancreatic cancer. In some preclinical experiments, it has only been preliminarily verified (Du et al., 2020).

However, some exploratory clinical studies have seen hope in specific contexts. For example, some studies have tried to combine PD-1 inhibitor, AG chemotherapy (gemcitabine + albumin paclitaxel) and radiotherapy to treat borderline resectable or locally advanced pancreatic cancer (Chen S. et al., 2023). Preliminary results indicate that this strong alliance strategy may improve surgical resection rates and objective remission rates. However, it should be noted that these results come from smaller phase II studies or cohort analyses, and their effectiveness still needs to be confirmed through large-scale randomized clinical trials.

There are also some new related preclinical studies: designing nano platforms to enhance the sensitivity of cancer cells to cytotoxic T cells through research (Huang et al., 2025). Regulating adenosine metabolism and breaking immune suppression (Fan et al., 2025). Innovative drug delivery systems can directly inhibit the invasion and metastasis of tumor cells and promote the infiltration of CD8+T cells at the tumor site (Argenziano et al., 2025). These preclinical studies have enormous potential.

This chapter systematically reviews the current status of gemcitabine clinical application in various cancer types, combination therapy strategies, and the challenges of drug resistance. As the table above clearly demonstrates, despite significant progress in combination therapy, drug resistance remains a major cause of treatment failure. Therefore, a deeper understanding of the mechanisms of gemcitabine resistance (as discussed in Chapter 4 below) is fundamental to developing effective strategies to overcome this resistance. Table 2 summarizes the various schemes.

**TABLE 2:** Summary of efficacy and safety for gemcitabine-based therapeutic regimens in key clinical trials.

Regimen	Cancer type	Line of therapy	Compare with	OS	PFS	ORR	Grade 3–4 toxicities
Gemcitabine Monotherapy	Metastatic Pancreatic Cancer	1st	5-FU	5.65 vs. 4.41 months	Not reported	Not reported	Not reported
FOLFIRINOX	Metastatic Pancreatic Cance	1st	Gemcitabine	11.1 vs. 6.8 months (HR 0.57; 95% CI 0.45–0.73; p < 0.001)	6.4 vs. 3.3 months (HR 0.47; 95% CI 0.37–0.59; p < 0.001)	31.6% vs. 9.4% (p < 0.001)	Neutropenia (45.7%), Febrile Neutropenia (5.4%)
AG (Nab-paclitaxel + Gemcitabine)	Metastatic Pancreatic Cance	1st	Gemcitabine	8.5 vs. 6.7 months (HR 0.72; p < 0.001)	5.5 vs. 3.7 months	23% vs. 7%	Neutropenia (38%), Fatigue (17%), Peripheral Neuropathy (17%)
AG (Modified Schedule)	Locally Advanced/Metastatic Pancreatic Cancer	1st	No randomized comparator	9.3 months	4.4 months	18.6%	Neutropenia (21%), Leukopenia (16%)
AG (JCOG1611)	Metastatic Pancreatic Cancer (Asian)	1st	mFOLFIRINOX	17.1 vs. 14.0 months	Not reported	Not reported	Not reported
Erlotinib + Gemcitabine	Advanced Pancreatic Cancer	1st	Gemcitabine	6.24 vs. 5.91 months (HR 0.82; p = 0.038)	Not reported	Not reported	Rash, Diarrhea
Gemcitabine + Cisplatin	Advanced Non-Small Cell Lung Cancer	1st	Cisplatin	Significantly prolonged (Specific value not reported)	8.2 months (Median PFS)	32% vs. 18%	Not reported
GCb (Gemcitabine + Carboplatin)	Advanced Non-Small Cell Lung Cancer	1st	No randomized comparator	Not reported	Not reported	Not reported	Not reported
Gemcitabine + Cisplatin (GC)	Bladder Cancer (Cisplatin-ineligible)	1st	MVAC	Similar efficacy, lower toxicity	Not reported	Not reported	Lower toxicity than MVAC
AG + Camrelizumab + Radiotherapy	Borderline Resectable/Locally Advanced Pancreatic Cancer	Neoadjuvant/1st	AG (Historical control)	Not reported	Not reported	Not reported	Not reported (Phase II study)
Gemcitabine + Durvalumab + Tremelimumab	Advanced Solid Tumors (subset of patients) ≥2nd	≥2nd	No randomized comparator	Not reported	Not reported	Efficacy in some patients	Not reported (Exploratory combination)
Olaparib (Maintenance)	BRCA-mutant Metastatic Pancreatic Cancer	1st-line Maintenance	Placebo	7.4 vs. 3.8 months (HR 0.60; p < 0.001)	As above (PFS is primary endpoint)	Not reported	Not reported
Olaparib + Cisplatin + Gemcitabine	BRCA/PALB2-mutant Pancreatic Cancer	1st	Cisplatin + Gemcitabine	Not reported	Not reported	48%	Not reported (Phase II study)
Decitabine + Gemcitabine	Advanced Pancreatic Cancer (low dCK expression)	≥2nd	Gemcitabine (Historical control)	Not reported	Not reported	34% vs. 12% (p = 0.013)	Myelosuppression (Alternating schedule required)
PEGPH20 (PEGylated hyaluronidase)	Pancreatic Cancer	Combination	No randomized data				
Disulfiram (ALDH inhibitor)	Pancreatic Cancer	Combination	No randomized data				
Vismodegib (Hedgehog inhibitor)	Pancreatic Cancer	Combination	No randomized data				
CD40 Agonist (e.g., Selicrelumab)	Pancreatic Cancer	Combination	No randomized data				
Oncolytic Virus (e.g., T-VEC)	Pancreatic Cancer	Combination	No randomized data				

## 3 Mechanism of action of gemcitabine

### 3.1 Molecular structure and activity characteristics

Gemcitabine is an analog of cytarabine and is used as a chemotherapy drug that inhibits pyrimidine nucleotide metabolism to treat testicuNCTlar cancer, breast cancer, ovarian cancer, non-small cell lung cancer, pancreatic cancer and bladder cancer. Gemcitabine is also a synthetic cytosine nucleoside derivative with strong radiosensitization and less toxic side (Danzi et al., 2023).

Gemcitabine has an anti-metabolic effect. After entering the cell, it is phosphorylated into an active triphosphate form under the action of intracellular kinases. This active metabolite can be mistakenly incorporated into the DNA chain by DNA polymerase, resulting in the blockage and termination of DNA chain extension, thereby inhibiting DNA synthesis (Dalin et al., 2019). Due to the blockage of DNA replication, the cell cycle is blocked, thereby inducing tumor cell apoptosis. This mechanism enables gemcitabine to exhibit a high activity in inhibiting tumor cell proliferation.

### 3.2 Metabolic mechanism

#### 3.2.1 Phosphorylation activation process

**FIGURE 2:**
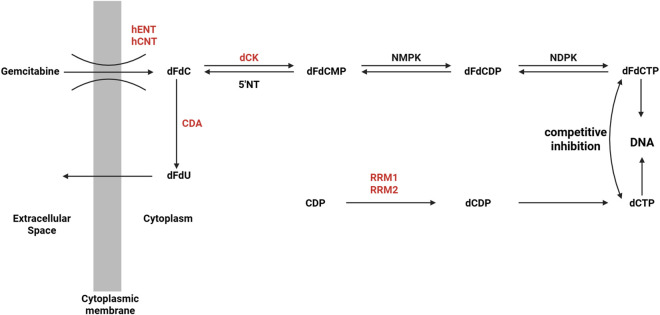
Gemcitabine cellular uptake and metabolism (Binenbaum et al., 2015).

Gemcitabine is a hydrophilic nucleoside analog. Due to its strong water solubility, it cannot directly penetrate the cell membrane and must rely on nucleoside transporters on the cell membrane (mainly including SLC29A1, SLC28A1 and SLC28A3) to transport it from the extracellular environment into the cell. After entering the cell, gemcitabine is catalyzed by deoxycytidine kinase (dCK) to generate monophosphate gemcitabine monophosphate (dFdCMP) (Chen J. et al., 2024). This phosphorylation reaction is the rate limiting step of drug activation. Subsequently, dFdCMP further accepts a phosphate group under the action of cytidine monophosphate kinase (CMPK) and is converted into gemcitabine diphosphate (dFdCDP) (Chen J. et al., 2024). Finally, under the catalysis of nucleoside diphosphate kinase (NDPK), dFdCDP is phosphorylated to the final active form gemcitabine triphosphate (dFdCTP). This step-by-step phosphorylation process ensures that gemcitabine participates in DNA synthesis in the form of an active molecule, thereby exerting its antitumor effects (Yang Q. et al., 2023). As shown in Figure 2, the above content is presented, the factors in red represent clinically significant participants inducing gemcitabine resistance (Binenbaum et al., 2015).

#### 3.2.2 Competitive inhibition of deoxycytidine metabolism

There is a competitive interaction between gemcitabine and deoxycytidine during the re-metabolism process, which is mainly reflected in the competitive relationship in cell uptake, phosphorylation activation and nucleoside metabolic pathways. Since both gemcitabine and deoxycytidine are pyrimidine nucleoside compounds, both rely on the same nucleoside transporters (such as SLC29A1, SLC28A1and SLC28A3) to enter cells (Lafazanis et al., 2024). When the concentration of deoxycytidine in the extracellular environment is high, it will compete with gemcitabine for binding sites, thereby affecting uptake and utilization, and reducing the concentration of gemcitabine in cells.

Similarly, deoxycytidine competitively inhibits gemcitabine and deoxycytidine kinase, competing with gemcitabine’s active triphosphorylation product (dFdCTP) for incorporation into the nascent DNA chain, resulting in a reduction in the cytotoxic effect of gemcitabine. Therefore, when the above two drugs are used in combination, attention should be paid to the order of administration and dosage adjustment.

### 3.3 Antitumor mechanism

#### 3.3.1 Inhibition of DNA synthesis

The active triphosphorylation product of gemcitabine (dFdCTP) can compete with deoxycytidine triphosphate (dCTP) for incorporation into nascent DNA chains. Since dFdCTP lacks a normal 3′-OH group, its incorporation terminates DNA chain extension and ultimately affects DNA synthesis (O et al., 2024; Plunkett et al., 1995). This mechanism has been clearly verified by biochemical methods such as radiolabeling experiments (^3^H-dFdCTP incorporation localization) and gel electrophoresis (fixed length DNA fragments) (Mini et al., 2006). This DNA damage can trigger cell cycle S phase arrest (through the ATR Chk1 signaling pathway) and induce apoptosis (such as mitochondrial pathway Bax/Bak activation). Clinical data show that the ability of dFdCTP accumulation in tumor cells of patients with PDAC is significantly correlated with prolonged survival (Khatri et al., 2014), highlighting the molecular basis of its treatment response.

#### 3.3.2 Induction of cell cycle arrest

The effect of gemcitabine on DNA synthesis results in chain termination. This “chain termination” effect directly induces DNA replication stress, prompting cells to arrest in the S phase to initiate DNA repair mechanisms or enter programmed cell death (Guenther et al., 2023).

Gemcitabine in its diphosphate form gemcitabine diphosphate (dFdCDP) inhibits ribonucleotide reductase ribonucleotide reductase (RNR), a key enzyme in the synthesis of deoxyribonucleotides (dNTPs). Inhibition of RNR leads to a significant decrease in intracellular dNTP levels, further impeding the DNA replication and repair process (Pereira et al., 2004). This dNTP deficient state also activates the cell’s DNA damage response, leading to cell cycle arrest in the S phase or G1/S transition phase.

Blocked DNA replication and insufficient dNTP supply trigger the DNA damage response, which in turn activates the S phase checkpoint (Li et al., 2024). Checkpoint signals prevent further replication of damaged DNA by activating related regulatory proteins, such as Checkpoint Kinase 1(CHK1), Checkpoint Kinase 2(CHK2), and P53, thereby arresting cell cycle progression. This mechanism causes cells to temporarily remain in the S phase in an attempt to repair damaged DNA; if repair fails, apoptosis may occur.

#### 3.3.3 Induction of cell apoptosis

Gemcitabine induced apoptosis primarily arises from cell cycle arrest triggered by DNA replication stress. When DNA synthesis is prematurely terminated due to the “chain termination” effect of gemcitabine incorporation, cells activate intrinsic DNA damage response (DDR) pathways to maintain genomic integrity. However, excessive replication stress surpasses repair capacity and consequently drives the activation of apoptosis related signaling cascades.

The diphosphate metabolite of gemcitabine (dFdCDP) functions as a potent inhibitor of ribonucleotide reductase (RNR), thereby suppressing the biosynthesis of deoxyribonucleotides (dNTPs). The resulting depletion of intracellular dNTP pools leads to severe defects in DNA replication, exacerbation of DNA strand breaks, and the initiation of programmed cell death (Ferreira et al., 2019). DNA damage and nucleotide insufficiency further activate cell cycle checkpoints and regulatory mediators (Salimi et al., 2020), amplifying the pro-apoptotic signal. At the mitochondrial level, increased outer membrane permeability facilitates the release of cytochrome c into the cytoplasm, which in turn triggers caspase activation and execution of apoptosis. In parallel, gemcitabine induced DNA damage may stimulate stress responsive signaling cascades such as the MAPK/JNK pathway that fine tune the balance between cell survival and death. Under conditions of overwhelming genotoxic stress, pro-apoptotic signaling predominates, ultimately committing the cell to apoptosis.

## 4 Mechanism of resistance to gemcitabine

### 4.1 Definition and current status of drug resistance

Drug resistance refers to the genetic or adaptive changes in tumor cells that reduce the killing effect of chemotherapy drugs, ultimately leading to treatment failure. According to the differences in the time and mechanism of drug resistance, it can be divided into two categories:

One type is primary resistance: tumor cells have inherent resistance characteristics before treatment, commonly found in tumors with wild type DNA repair genes (such as BRCA1/2) or metabolic enzyme defects (Holohan et al., 2013; Li et al., 2025). Another type is acquired resistance, which gradually evolves during treatment through genetic mutations (such as RRM1 amplification), epigenetic remodeling (such as lncRNA regulation), or microenvironmental adaptation (such as hypoxia induction) (Yang et al., 2021).

In recent years, the application of single-cell multi omics technologies such as scRNA seq and spatial metabolomics has provided a new perspective for analyzing the dynamic evolution of drug-resistant clones (Roehrig et al., 2024).

### 4.2 Resistance mechanisms at the molecular level

#### 4.2.1 Abnormal expression of nucleoside metabolizing enzymes and individual differences

Dysfunction of deoxycytidine kinase (dCK): dCK is the rate limiting enzyme activated by gemcitabine, catalyzing its phosphorylation into the active form gemcitabine triphosphate (dFdCTP) (Wang et al., 2022). Clinical studies have shown that dCK gene polymorphism (such as rs11158728 SNP) can reduce the enzyme (Dash et al., 2024), and the objective response rate (ORR) of pancreatic cancer patients with low activity genotype decreases (Hatori et al., 2024). CRISPR screening confirmed that dCK knockout can increase the half maximal inhibitory concentration (IC50) of gemcitabine (Yang et al., 2022).

Overexpression of ribonucleotide reductase (RNR) subunits: RNR is composed of RRM1 (regulatory subunit) and RRM2 (catalytic subunit), responsible for synthesizing dNTP (Zuo et al., 2024). The metabolite dFdCDP of gemcitabine can competitively inhibit RNR, but overexpression of RRM1/RRM2 can restore dNTP concentration to >150μM, significantly reducing drug sensitivity (p < 0.001) (Shu et al., 2020). Activation of the MAPK/ERK pathway phosphorylates the RRM2 Ser20 site, enhancing its stability and promoting drug resistance (Zhan et al., 2021).

Functional deficiency of nucleoside transporter hENT1: hENT1 mediates cellular uptake of gemcitabine, and abnormal glycosylation modifications can lead to membrane localization disorders and reduce drug influx (Chakraborty et al., 2018). Intervention strategies include developing hENT1 independent prodrugs (such as liposomal gemcitabine) or combining HDAC inhibitors to restore transporter expression (Xi et al., 2020).

**FIGURE 3:**
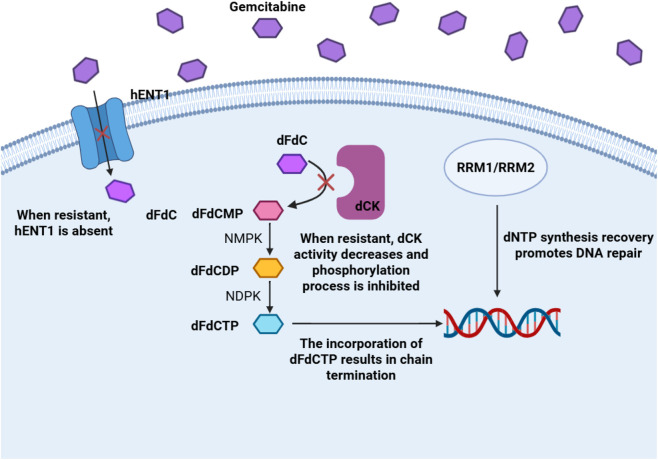
Metabolic pathways, mechanisms of action, and resistance molecular pathways of gemcitabine.

As shown in Figure 3, for ease of understanding, we visualize this process to elucidate the generation of drug resistance mechanisms at the molecular level.

#### 4.2.2 ABC transporter mediated drug efflux

ABC transporters utilize ATP hydrolysis to efflux gemcitabine and its metabolites from cells, thereby reducing intracellular drug concentrations and contributing to resistance.

P-glycoprotein (P-gp, ABCB1) increases in many gemcitabine-resistant cell lines and pancreatic cancer samples, and molecular docking plus lab tests show it can bind and pump out gemcitabine. It uses ATP from mitochondria for this, and blocking P-gp with diltiazem brings back gemcitabine’s toxicity (Gu et al., 2022).

Multidrug resistance protein 1 (MRP1, ABCC1) also rises in resistant cells, and it can pump out gemcitabine and its deaminated form dFdU, so drug levels stay low inside cells. Blocking MRP1 with MK-571 partly reverses resistance and makes cells more sensitive to gemcitabine (Chen DQ. et al., 2024; Hopper-Borge et al., 2009).

Multidrug resistance protein 7 (MRP7, ABCC10) can pump out gemcitabine. When we put MRP7 into HEK293 or mouse embryonic fibroblast (MEF) cells, their gemcitabine IC50 goes up by about five times. And unlike MRP1/2, MRP7 does not need glutathione to pump out the drug (Chen DQ. et al., 2024).

**FIGURE 4:**
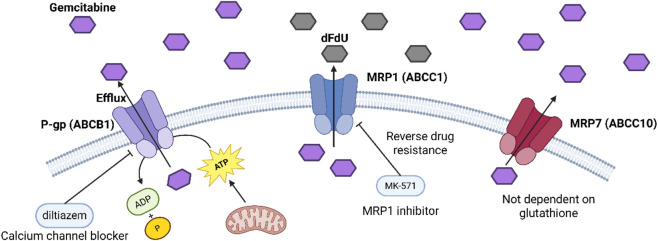
Mechanism of action of ABC transporter inhibitor.

Figure 4 shows the chemical structures of two ABC transporter inhibitors: diltiazem and MK-571. These compounds were used in the study to block the efflux of gemcitabine mediated by ABC transporters such as P-gp and MRP1, thereby restoring tumor cell sensitivity to drugs and reversing drug resistance.

#### 4.2.3 Enhancement of DNA repair mechanisms

Tumor cells resist the killing effects of chemotherapeutic drugs by enhancing their DNA repair ability, which has become an important aspect of drug resistance. Among them, the activation of two major pathways, base excision repair (BER) and homologous recombination repair (HR), is one of the key factors (Helleday et al., 2008).

First, base repair refers to the cell repair of base damage caused by endogenous or exogenous factors (such as chemotherapy drugs). This process starts with DNA glycosidase recognizing and removing the damaged base to form an abasic site (AP site), AP endonuclease cutting the abasic site, DNA polymerase filling the missing base, and DNA ligase filling the gap. Chemotherapeutic drugs such as gemcitabine induce cell apoptosis by introducing DNA chain extension errors or forming abnormal DNA structures. However, if BER activity in tumor cells is significantly enhanced, these damages can be repaired rapidly, thereby reducing drug-induced cell death (Piscone et al., 2025).

Secondly, homologous recombination repair is another high-fidelity DNA repair mechanism, which is mainly used to repair double strand breaks. Homologous recombination repair requires homologous chromosomes as templates and repairs broken DNA through a series of precise pairing and exchange steps. DNA damage caused by chemotherapy drugs (such as drug action leading to replication fork collapse) often produces double-strand breaks. If tumor cells activate the homologous recombination repair pathway by upregulating key repair proteins (such as BRCA1/2, RAD51, etc.), they can efficiently repair these serious damages and thus escape chemotherapy induced cell apoptosis (Casolino et al., 2021).

In summary, this mechanism plays an important role in chemotherapy resistance, revealing that inhibiting specific repair pathways (such as using PARP inhibitors to interfere with BER) may be a new strategy to overcome resistance (Fatteh et al., 2024; Kawamoto et al., 2024).

### 4.3 Relationship between tumor microenvironment and drug resistance

The complexity and heterogeneity of the tumor microenvironment provide multiple protection mechanisms for tumor cells to escape drug attack and is a major culprit of tumor resistance.

Tumor vascular barrier function: Tumors often have abnormally constructed vascular systems inside them. These blood vessels have strong permeability but disordered structure and uneven distribution. Abnormal tumor blood vessels form a barrier that limits the uniform distribution and effective delivery of anticancer drugs, making it difficult for drugs to fully penetrate the entire tumor tissue (Druzhkova et al., 2022; Zhang et al., 2025). In addition, the vascular barrier can also lead to uneven local drug concentrations, with drug concentrations in some areas being too low, providing tumor cells with an opportunity to escape the effects of the drug. When the drug cannot reach sufficient intracellular concentrations, the cells may survive through intrinsic drug resistance mechanisms and gradually evolve into drug-resistant tumors.

Effect on drug resistance: Hypoxia and acidic conditions are common in the tumor microenvironment. The hypoxic state not only directly reduces the activity of certain drugs, but also activates the expression of a series of genes related to drug resistance by inducing upregulation of hypoxia-inducible factor (HIF) (Cabanos and Hata, 2021; Belisario et al., 2020). HIF can promote changes in cell metabolism and activate pro-survival signaling pathways, thereby increasing the tolerance of tumor cells to chemotherapeutic drugs. At the same time, hypoxia can also promote epithelial-mesenchymal transition (EMT) in tumor cells, further enhancing their invasiveness and drug resistance (Hapke and Haake, 2020).

The efficient glycolysis of tumor cells leads to an acidic environment in the tumor microenvironment. This acidic condition affects the permeability and stability of drugs and reduces the effective concentration of drugs in cells (Marchand et al., 2023). In addition, the acidic environment may also change the charge distribution of the cell membrane, interfere with the transmembrane transport of drugs, and ultimately inhibit the efficacy of drugs.

**FIGURE 5:**
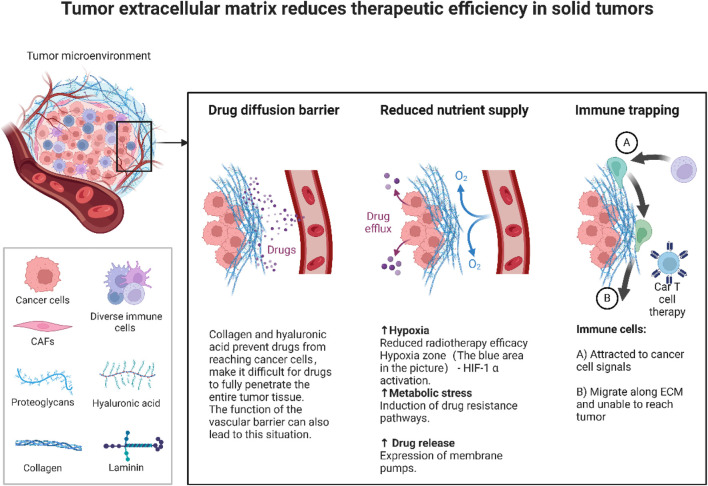
Tumor microenvironment barrier.

Figure 5 illustrates how the solid tumor microenvironment (TME) drives treatment resistance. A dense extracellular matrix (ECM), rich in collagen and hyaluronic acid, forms a physical barrier that restricts drug penetration and distribution. A dense extracellular matrix (ECM),rich in collagen and hyaluronic acid, forms a physical barrier that restricts drug penetration and distribution. Concurrently, abnormal vasculature and ECM accumulation foster hypoxic and acidic conditions, stabilizing HIF-1α. This transcription factor activates pro-survival pathways, induces epithelial mesenchymal transition (EMT), and upregulates drug efflux pumps, thereby promoting drug resistance. Additionally, the ECM impedes immune cell infiltration, compromising T cell mediated cytotoxicity and limiting the efficacy of immunotherapies.

### 4.4 Epigenetic regulation of drug resistance

In the epigenetic regulatory network of gemcitabine resistance, long noncoding RNA (lncRNA) mediated RRM2 regulation and dynamic regulation of drug-metabolizing enzymes by m^6^A (N6-methyladenosine) methylation modification are two key mechanisms that together constitute a complex resistance network:

In the mechanism of gemcitabine resistance, oncogenic lncRNAs (such as MALAT1 and NEAT1) significantly promote the expression of ribonucleotide reductase M2 (RRM2) through epigenetic regulation (Wang et al., 2021). MALAT1 relieves the inhibition of RRM2 by adsorbing miR-26a/b and enhancing the stability of RRM2 mRNA (Yang et al., 2024),while NEAT1 indirectly activates RRM2 transcription by sequestering RNA binding proteins (such as SFPQ) (Hu et al., 2021). Overexpression of RRM2 accelerates deoxynucleotide synthesis, competitively antagonizes the DNA intercalation of gemcitabine, and enhances DNA repair capacity, thereby weakening the efficacy of the drug (Zuo et al., 2024). Targeted silencing of these lncRNAs (such as using antisense oligonucleotides) can reduce RRM2 levels and restore chemotherapy sensitivity.

m^6^A methylation modification regulates key enzymes in gemcitabine metabolism through the dynamic balance of METTL3 (methyltransferase-like 3) and FTO (Fat mass and obesity-associated protein, demethylase) (Du et al., 2025). METTL3 mediated m^6^A modification enhances the mRNA stability of deoxycytidine kinase (dCK) to promote gemcitabine activation (Chen J. et al., 2023), but on the other hand, it upregulates RRM2 expression, leading to drug resistance (Lin K. et al., 2023),while FTO inhibits RRM2 expression by removing m^6^A modification, but may also reduce dCK activity (Wei et al., 2022). In addition, m^6^A and lncRNA have cross-regulation (such as METTL3 modification of MALAT1 to enhance its stability, and FTO regulates NEAT1 function) (Mao et al., 2022), forming a synergistic drug resistance network. Targeted METTL3 inhibitors or FTO agonists can partially reverse drug resistance (Lin C. et al., 2023), but it is necessary to balance its bidirectional effects on dCK and RRM2. In the future, multi-node interventions will be needed to optimize efficacy.

**FIGURE 6:**
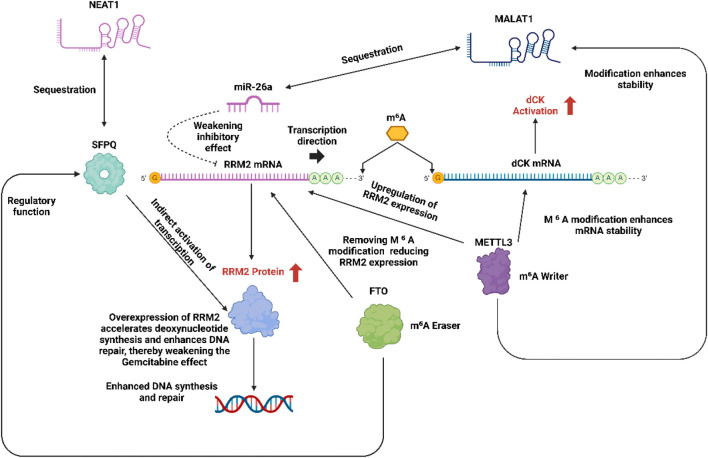
LncRNA mediated RRM2 regulation in the mechanism of gemcitabine resistance and bidirectional regulation of dCK and RRM2 by m^6^ A methylation.

Figure 6 illustrates the epigenetic and post-transcriptional regulatory network contributing to gemcitabine resistance through modulation of RRM2 and dCK expression. Long noncoding RNAs (NEAT1 and MALAT1) act as molecular sponges, sequestering key regulatory molecules NEAT1 indirectly activates RRM2 transcription by binding SFPQ and suppressing miR-26a, while MALAT1 enhances dCK mRNA stability via m^6^A modification. The METTL3-mediated methylation of RRM2 and dCK mRNAs increases their stability and expression, whereas the FTO demethylase counteracts this process by removing m^6^A marks. Elevated RRM2 expression promotes deoxynucleotide synthesis and DNA repair, ultimately diminishing gemcitabine cytotoxicity.

#### 4.4.1 Dynamic equilibrium and drug resistance of m^6^ A modification

m^6^A modification dynamically regulates RNA metabolism through the “writer-eraser-reader” ternary system:

METTL3/WTAP complex recognizes the GGACU motif of RRM2 mRNA, adds an m^6^A mark to its 3′UTR, recruits YTHDF2 reader, enhances mRNA stability and promotes translation (Zaccara et al., 2019).

FTO removes the m^6^A mark of RRM2 mRNA through demethylation, blocks YTHDF2 binding, and causes mRNA to be degraded by the CCR4-NOT complex.

Bidirectional regulatory contradiction:

METTL3 simultaneously mediates the m^6^A modification of the 5′UTR of dCK mRNA, promotes nuclear export and translation through YTHDC1, and enhances gemcitabine activation, while FTO demethylates dCK, reducing its stability, forming a balanced antagonism that promotes drug resistance/sensitization.

#### 4.4.2 Cross regulation of lncRNA and m^6^A

MALAT1-m^6^A positive feedback: METTL3 adds m^6^A modification to the Exon 6 region of MALAT1, recruits IGF2BP1 protein, enhances its stability and promotes nuclear retention, maintaining its adsorption capacity for miR-26a (Gu N. et al., 2024).

NEAT1-FTO negative regulation:FTO demethylates the m^6^A site of NEAT1, destroys its binding to HNRNPK, leads to NEAT1 degradation, releases the isolation of SFPQ, and inhibits RRM2 transcription (Knutsen et al., 2022).

### 4.5 Abnormalities of classical metabolic pathways

In the mechanism of gemcitabine resistance, the functional defect of the nucleoside transporter hENT1 (human equilibrative nucleoside transporter 1) is the core link of the abnormality of the classical metabolic pathway. Recent studies have found that abnormal glycosylation modification of hENT1 protein may be one of the important reasons for its loss of function (Gil-Chinchilla et al., 2024).

In the mechanism of gemcitabine resistance, the functional defect of nucleoside transporter hENT1 is the core factor of metabolic pathway abnormality. Its abnormal glycosylation modification (such as lack of N-glycosylation or insufficient O-glycosylation) can lead to hENT1 membrane localization disorder (endoplasmic reticulum degradation or intracellular retention) or decreased stability (accelerated protease degradation) (Chakraborty et al., 2018), thereby reducing gemcitabine influx and activation. Competitive binding of glycoproteins (such as MUC1) in the microenvironment or glycosylation reprogramming induced by inflammatory factors (such as IL-6) (such as ST6GAL1 mediated sialylation) further interferes with hENT1 function (Jin et al., 2023). Intervention strategies include targeting glycosylation enzymes (such as inhibiting ST6GAL1), developing hENT1 independent prodrugs (such as liposome-encapsulated gemcitabine) or combining epigenetic regulators (HDAC inhibitors) (Li et al., 2019; Perera et al., 2022), but clinical translation needs to address the complexity of multi-pathway coordinated regulation and the challenges of standardized detection of glycosylation markers.

## 5 Strategies for addressing resistance to gemcitabine

### 5.1 New drug delivery systems and precision drug delivery

In recent years, researchers have developed a variety of new drug delivery systems to optimize the antitumor activity of gemcitabine, which has limitations such as strong hydrophilicity, rapid metabolism in the body, and low intracellular concentration. Nano drug delivery systems use nanoscale carriers (such as liposomes, polymers, albumin, etc.) to improve the stability of gemcitabine, prolong circulation time, and increase accumulation through targeted release at tumor sites. For example, pH-sensitive nanoparticles achieve passive targeting through the EPR effect (enhanced permeability and retention effect) in pancreatic cancer models, significantly enhancing drug delivery and reducing toxicity (Yetisgin et al., 2020). In addition, albumin nanoparticles can bypass the hENT1-dependent uptake pathway due to their natural targeting (binding to the tumor microenvironment through SPARC protein) and unique transmembrane mechanisms (such as gp60 receptor mediated transport), directly increasing the concentration of gemcitabine in resistant tumor cells, and have shown advantages in overcoming hENT1-deficient resistance in pancreatic cancer and bladder cancer models (Bartkowski et al., 2024; Liu et al., 2025).

Among the carrier types, liposomes can reduce immune clearance and prolong circulation time through PEGylation modification (Aparicio-Lopez et al., 2024), while liposomes modified with targeting ligands (such as folic acid or EGFR antibodies) can further achieve active targeting (Petrikaite et al., 2023).

Polymer carriers (such as PLGA, chitosan) can precisely release drugs through stimulus responsive design (pH/temperature sensitivity) and can enhance tumor enrichment through surface targeting molecules (such as hyaluronic acid) (Danhier et al., 2012; Wang et al., 2017). In addition, the development of local sustained release preparations provides new ideas for specific indications. For example, gemcitabine thermosensitive hydrogels or microspheres are used for intraperitoneal perfusion to treat peritoneal metastasis, which can form a drug sustained release reservoir in the peritoneal cavity, prolong local exposure time and reduce systemic toxicity (Xu et al., 2016). Preclinical studies have shown that it significantly inhibits tumor dissemination in ovarian cancer and gastric cancer peritoneal metastasis models (Xu et al., 2016). Other innovative platforms such as gold nanoparticles (photothermal controlled release) (Vines et al., 2019), magnetic nanoparticles (magnetic targeting) and microfluidic chip systems integrate targeting, imaging and controlled release functions to promote the development of precision therapy. The diversification of these delivery systems provides an important strategy for breaking through gemcitabine resistance and expanding clinical applications.

Numerous preclinical studies have demonstrated that innovative design can significantly overcome the bottleneck of low EPR effect caused by dense matrix of pancreatic cancer, and improve the tumor enrichment and penetration efficiency of nanoparticles. The core strategy includes: 1. Matrix remodeling, such as using acid sensitive macromolecular nanoparticles to target and clear CAFs, reducing matrix density, thereby enhancing subsequent nanomedicine delivery (Zanganeh et al., 2016). 2. Actively targeted, the nanoparticles were modified with membrane penetrating peptides such as iRGD, and their matrix penetration ability in the pancreatic cancer model was improved by up to 300% by specifically binding to neurociliain-1 (NRP-1) (Sugahara et al., 2010). 3、 For size and surface engineering, the study found that the non PEG modified polyphosphate nanoparticles (NoPEG NPDox) can be more effectively absorbed by pancreatic cancer cells than the traditional PEG particles, showing a stronger killing effect (Eom et al., 2018). 4. Intelligent response release, such as building pH responsive iCluster nano system, which dissociates from about 100 nm to 5 nm micro particles in an acidic microenvironment, significantly enhancing the deep penetration ability of pancreatic cancer matrix (Huo et al., 2014). The effectiveness of these strategies has been validated through techniques such as live imaging of small animals, quantitative analysis of radiolabeled PET, and microscopic imaging of tumor tissue sections. However, these conclusions are still mainly based on mouse models, and their feasibility and efficacy for clinical translation still need further validation.

**FIGURE 7:**
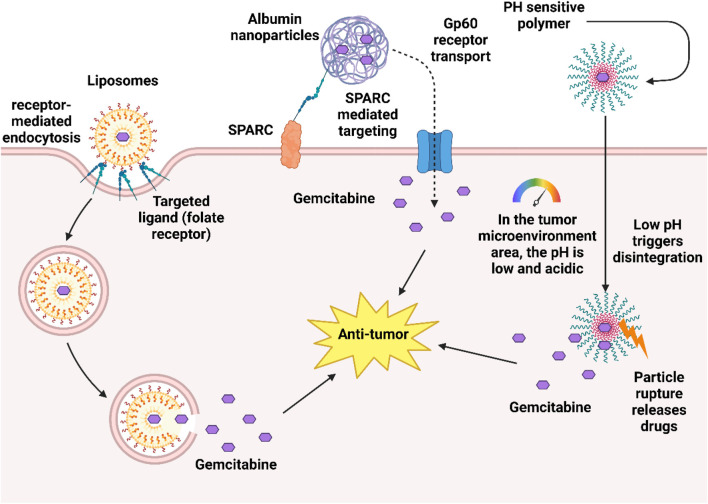
Nanocarrier designs to overcome gemcitabine resistance.

Figure 7 illustrates a dual-targeting nanoparticle drug delivery system designed for the tumor microenvironment. The system employs albumin nanoparticles loaded with gemcitabine, which exploit the SPARC protein and gp60 receptor mediated pathways for preferential tumor cell uptake. Concurrently, pH sensitive polymeric components are engineered to destabilize and rupture in the acidic tumor milieu, ensuring precise and controlled drug release at the target site.

### 5.2 Combination therapy strategies

In recent years, the combination therapy strategy for gemcitabine resistance has made important breakthroughs, mainly focusing on the following directions:

Targeted inhibition of RRM1 and synthetic lethality: Inhibitors of the large subunit of ribonucleotide reductase RRM1 (such as COH29) enhance efficacy through dual mechanisms: (1) inhibiting dNTP biosynthesis, (via RNR/RRM1 suppression), leading to depletion of intracellular dNTP pools notably with deoxycytidine triphosphate (dCTP) often being suppressed in multiple studies, though the magnitude of decrease varies by cell type, dose, and duration (Huff et al., 2022). (2) blocking the homologous recombination repair (HRR) pathway, forming a synthetic lethal effect with gemcitabine induced DNA damage. Preclinical studies have shown that COH29 co-treatment can reduce tumor volume in pancreatic cancer patient-derived xenograft (PDX) models by 72% and delay the emergence of drug-resistant clones (3.2-fold delay in emergence) (Chen et al., 2015). Notably, this strategy can overcome the compensatory upregulation of RRM2 mediated by lncRNA MALAT1.

Epigenetic regulation combined therapy: DNA methyltransferase inhibitor decitabine reverses drug resistance through dual effects: (1) removing the hypermethylation of CpG islands in the promoter region of the dCK gene and restoring the drug activation ability (Paweł and Maria Małgorzata, 2022). (2) upregulating hENT1 expression (2.7-fold increase) and enhancing gemcitabine transmembrane transport (Konaté et al., 2024). A phase II clinical trial (NCT03247088) demonstrated that in advanced pancreatic cancer patients with low dCK expression, the decitabine combination regimen achieved an objective response rate (ORR) of 34%, significantly higher than the 12% observed in the gemcitabine monotherapy group (p = 0.013). Moreover, the study underscored the need to closely monitor for cumulative toxicities, such as bone marrow suppression, and recommended an alternating dosing schedule administering gemcitabine on days 1 and 8, and decitabine on days 5–7 to mitigate these risks (Hindson, 2022).

Synergistic immunotherapy strategy: Gemcitabine releases damage-associated molecular patterns (damage-associated molecular patterns, DAMPs) such as HMGB1 by inducing immunogenic cell death (immunogenic cell death, ICD), producing a synergistic effect with PD-1 inhibitors (Liu H. et al., 2024). In a pancreatic cancer mouse model, CD8^+^ T cell infiltration in the combined treatment group increased by 4.1 times, and the complete tumor regression rate increased to 42% (8% in the single drug group) (Hayashi et al., 2020). The KEYNOTE-202 clinical trial showed that this regimen prolonged the median overall survival (mOS) of patients with metastatic pancreatic cancer to 14.7 months (9.8 months in the single drug group, HR = 0.62) (Bockorny et al., 2021).

Nano co-delivery system: Albumin nanoparticles co-loaded gemcitabine and MEK inhibitors, increasing the tumor/plasma drug concentration ratio to 9.8:1, significantly inhibiting the activation of the MAPK pathway (Liu D. et al., 2024).

Multi-target combination: The combination of EGFR/VEGF dual-targeting inhibitors showed a synergistic effect in the KRAS mutation model (combination index CI = 0.32) (Yao et al., 2022).

### 5.3 Remodeling of the immune microenvironment

In the immune combination strategy to address gemcitabine resistance, CD40 agonist activation of antigen presentation and oncolytic virus-induced “viral mimicry” to enhance immunogenic cell death (ICD) are two key directions, which can enhance the efficacy of chemotherapy and reverse resistance by reshaping the immune microenvironment:

CD40 agonists (such as Selicrelumab) promote tumor antigen presentation and T cell activation by activating CD40 receptors on the surface of dendritic cells (Vonderheide, 2020), and form an “antigen release-presentation-response” closed loop when combined with gemcitabine. Gemcitabine induces tumor antigen release (such as HMGB1) and reduces myeloid-derived suppressor cells (MDSCs), while CD40 agonists enhance the expression of co-stimulatory molecules (CD80/86), drive CD8^+^ T cell infiltration and inhibit Treg function (Le and Jaffee, 2013). Preclinical studies have shown that they can activate type I interferon responses mediated by the STING pathway (such as pancreatic cancer models). Despite the risk of cytokine release syndrome (CRS), this combination strategy provides a new direction for reversing the immunosuppressive microenvironment and overcoming drug resistance (Suzuki et al., 2024).

Oncolytic viruses (such as T-VEC) selectively lyse tumor cells to release DAMPs (such as CRT, HMGB1) and viral nucleic acids, triggering strong immunogenic cell death (ICD) and activating the cGAS-STING pathway, inducing a “viral mimic” state to enhance antitumor immunity (Mohseni et al., 2021). Its combination with gemcitabine can amplify the ICD effect of chemotherapy (Kajiwara et al., 2023), while reshaping the microenvironment (reducing M2 macrophages, recruiting NK cells) and overcoming DNA repair resistance mediated by RRM1 overexpression (such as triple-negative breast cancer models). In the future, it is necessary to optimize viral vectors (such as carrying gemcitabine prodrug genes) and combination timing, and screen the beneficiary population based on PD-L1 expression or T cell infiltration to balance the potential risk of systemic infection and achieve precision immunochemotherapy.

### 5.4 Biomarker-guided treatment

In the precision treatment strategy for gemcitabine resistance, biomarker guided treatment based on dynamic monitoring of RRM2 copy number based on ctDNA and multi-omics prediction models (such as the EXPLOR trial) provides a new paradigm for individualized intervention:

Gemcitabine resistance, RRM2 gene copy number amplification drives resistance by accelerating deoxyribonucleotide triphosphate (dNTP) synthesis and enhancing DNA repair, while ctDNA-based liquid biopsy technology (such as ddPCR/NGS) can dynamically monitor the level of RRM2 amplification and track the evolution of resistant clones in real time. Through high-frequency ctDNA detection, clinicians can detect resistance trends (such as RRM2 copy number doubling) earlier than imaging, and guide the adjustment of treatment plans (such as switching to RRM2 inhibitor 3-AP or combined with METTL3 inhibitor) to avoid ineffective chemotherapy. Studies have confirmed that dynamic monitoring of ctDNA combined with intervention strategies can delay the progression of resistance (Cescon et al., 2020).

The EXPLOR trial integrated the genome (RRM1/2 mutation), transcriptome (dCK/hENT1 expression), epigenome (dCK methylation) and immune microenvironment characteristics to construct a machine learning model (AUC = 0.89) to predict gemcitabine sensitivity. The key marker combination (such as RRM2 amplification + dCK hypomethylation + CD8^+^ T cell low infiltration) can accurately identify resistant patients. After the dynamic multi-omics data iterative optimization model, it can guide stratified treatment (such as switching resistant patients to gemcitabine + PD-1 inhibitor + oncolytic virus triple regimen). Prospective trials have shown that it prolongs the median PFS of resistant patients by 3.2 months. In the future, the sensitivity of ctDNA detection, multi-omics standardization and cost barriers need to be resolved to promote the clinical application of the “monitoring-prediction-intervention” closed loop.

## 6 Cutting-edge technologies, tools and achievements in gemcitabine-related research

### 6.1 Application of single-cell sequencing in gemcitabine research

Single-cell RNA sequencing (scRNA-seq) technology can analyze the heterogeneity within tumor cells (Qin et al., 2023; Chen C. et al., 2022), helping researchers identify a small number of cell subpopulations that develop resistance during gemcitabine treatment. By sequencing the transcriptome of a single cell, it is possible to accurately capture changes in the expression of key resistance genes and reprogramming of signaling pathways within the cell, thereby revealing the molecular mechanism of gemcitabine resistance. For example, single-cell sequencing technology revealed the clonal evolution path of gemcitabine resistance by analyzing the genomic, transcriptomic, and epigenetic heterogeneity of tumor cells before and after treatment (El-Tanani et al., 2025). Before treatment, the tumor already has pre-resistant subclones (such as those carrying RRM2 copy number gain, low dCK expression, or activated DNA repair pathways) (Kyrochristos et al., 2019). After gemcitabine treatment, sensitive clones are eliminated, while pre-existing or newly emerged resistant clones (such as TP53 mutation, RRM2 amplification) dominate through adaptive reprogramming (such as activation of the ATF4 stress pathway, enhanced glycolysis) (Luo and Liang, 2023). At the same time, CAFs in the tumor microenvironment activate STAT3 signaling by secreting IL-6, promoting the maintenance of stemness and immune escape of resistant clones, driving clonal evolution (Kwon et al., 2025). Single-cell multi-omics analysis further identified key resistance-driving events, such as reduced chromatin accessibility of the dCK promoter (confirmed by scATAC-seq) (Fang et al., 2024) and cell cycle deregulation caused by CDKN2A loss (Buj et al., 2025). These findings provide a basis for dynamic monitoring (such as tracking the proportion of RRM2 amplified subclones) and targeted intervention (such as combined ATF4 inhibitors or IL-6/JAK blockers) (Wei et al., 2021; Thuya et al., 2025). Although single-cell technology faces challenges such as high cost and complex data analysis, its integration with liquid biopsy (ctDNA) and spatial transcriptome is expected to achieve early elimination of drug-resistant clones and personalized treatment, and promote the clinical application of the “spatiotemporal evolution-targeted intervention” precision model.

In addition, single-cell sequencing can also be used to monitor the dynamic changes of immune cells in the tumor microenvironment, providing a basis for combined immunotherapy.

Delineates the dynamic evolution of intratumoral heterogeneity under therapeutic pressure. It illustrates how pre-existing resistant subclones (e.g., with RRM2 amplification or dCK methylation) are selectively enriched by treatment, while sensitive clones (with high hENT1/dCK expression) are suppressed. The tumor microenvironment (TME), including cancer-associated fibroblasts (CAFs), supports this clonal selection. Monitoring techniques such as ctDNA profiling and single-cell sequencing can track these dynamics, often detecting resistant clones prior to radiographic progression.

**FIGURE 8:**
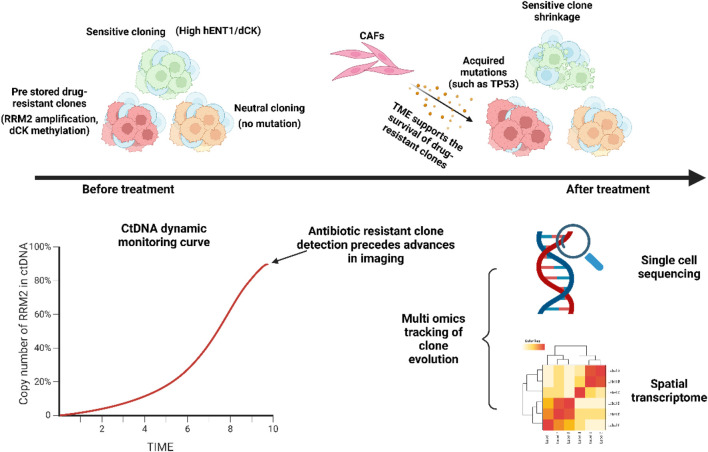
Dynamic resistance evolution model.

Figure 8 delineates the dynamic evolution of intratumoral heterogeneity under therapeutic pressure. It illustrates how pre-existing resistant subclones (e.g., with RRM2 amplification or dCK methylation) are selectively enriched by treatment, while sensitive clones (with high hENT1/dCK expression) are suppressed. The tumor microenvironment (TME), including cancer-associated fibroblasts (CAFs), supports this clonal selection. Monitoring techniques such as ctDNA profiling and single-cell sequencing can track these dynamics, often detecting resistant clones prior to radiographic progression.

### 6.2 Artificial intelligence optimizes combination drug therapy

Artificial intelligence (DeepChem platform) optimizes the core functions and strategies of gemcitabine combination therapy. The DeepChem platform integrates multi-omics data (genomic, epigenetic, metabolomics) and drug interaction networks through deep learning models (such as neural networks) to accurately predict gemcitabine synergistic drug regimens (Torkamannia et al., 2023). For example, for tumors with high RRM2 expression, the model recommends combining with CDK4/6 inhibitors to block DNA repair (Seetharam et al., 2024), for patients with low hENT1 expression and drug resistance, albumin nanoparticles or HDAC inhibitors are preferred to restore drug uptake (Roca et al., 2022). The platform also uses reinforcement learning to optimize dosing timing (such as gemcitabine 48 h before PD-1 inhibitors (Eastman et al., 2021)) and dose adjustments (such as reducing gemcitabine dose by 20% when combined with METTL3 inhibitors), balancing efficacy and toxicity (such as the risk of bone marrow suppression).

Gemcitabine + ATR inhibitor regimen generated by DeepChem achieved an objective responRegarding the latest se rate (ORR) of 38% in platinum resistant bladder cancer, which is 2 times higher than the traditional regimen. The prediction accuracy of virtual clinical trials based on patient organoids reached 82% (Chen et al., 2021). However, the generalizability of the model is limited by data heterogeneity (such as cancer-specific bias), and the black box characteristics need to be combined with explainable AI (XAI) methods (such as SHAP values) to analyze mechanism associations (Abbasian et al., 2025).

### 6.3 Global research progress

Notable recent global research advances include the biomarker exploration of the GEMSTONE trial and the NCI-MATCH sub-program announced by ESMO in 2023 provides new directions for overcoming drug resistance:

The GEMSTONE trial evaluated a novel oral gemcitabine formulation (such as liposomes or prodrug forms) (Zhang et al., 2024), which improved bioavailability by optimizing the drug delivery system (Zhang et al., 2024) and bypassed the hENT1 dependent cellular uptake pathway (Xi et al., 2020). After intestinal absorption, the formulation enters the liver directly through the portal vein, reducing first pass metabolism and significantly increasing blood drug concentrations. Its advantages in overcoming drug resistance are: 1) Oral formulations are delivered through albumin binding or nanocarriers to avoid insufficient uptake caused by low hENT1 expression (D et al., 2025), and in pancreatic cancer models, the efficacy is 30% higher than that of intravenous formulations. It is combined with PARP inhibitors (such as olaparib) to prolong progression-free survival (PFS 8.1 vs. 5.3 months) for patients with DNA repair defects (such as BRCA mutations), and has strong combination potential (Chen et al., 2018).

The NCI-MATCH subprogram (NCI-Molecular Analysis for Therapy Choice) is a large scale precision medicine clinical trial officially launched by the National Cancer Institute (NCI) in August 2015 (O'Dwyer et al., 2023). It aims to match targeted therapeutic drugs for patients with advanced cancer based on the molecular characteristics of the tumor (such as gene mutation, amplification or fusion). The subprogram for exploring biomarkers related to the mechanism of gemcitabine resistance (such as EAY131-Y) was mentioned when the relevant results were announced at the 2023 ESMO conference. The NCI-MATCH subprogram revealed key biomarkers of gemcitabine resistance through multi omics analysis (whole exome sequencing, RNA seq) (Yang et al., 2021): RRM2 gene amplification (copy number ≥4) significantly increased the risk of single drug resistance (HR = 2.1), dCK promoter hypermethylation led to drug activation disorders, and low infiltration of CD8^+^ T cells indicated immune microenvironment suppression (Carpenter et al., 2020). Based on marker stratification, the study recommends a precise combination strategy: patients with RRM2 amplification combined with RRM1 inhibitors (COH29) or METTL3 inhibitors (STM2457), and patients with dCK silencing combined with decitabine and albumin nanoparticles, to overcome metabolic defects and restore drug sensitivity. However, the standardization and accessibility of biomarker testing remain the main challenges for clinical promotion. In the future, it is necessary to combine dynamic ctDNA monitoring with AI models to optimize stratified treatment and promote the implementation of individualized solutions.

## 7 Conclusion and thoughts

Gemcitabine, a deoxycytidine analogue with potent antineoplastic activity, remains a cornerstone of modern oncology. As a chain terminator that competitively incorporates into elongating DNA strands, this pyrimidine analog inhibits DNA synthesis and exhibits broad spectrum cytotoxicity against diverse solid tumors. Its clinical dominion spans pancreatic adenocarcinoma, non-small cell lung carcinoma, and urothelial cancers, where synergistic regimens with platinum derivatives, taxanes, molecular targeted agents, and immune checkpoint modulators have revolutionized therapeutic paradigms. Such combinatorial approaches aim to overcome the primary limitation of monotherapy: drug resistance.

However, gemcitabine resistance remains a formidable challenge in clinical oncology. Mechanistic investigations reveal a constellation of molecular mechanisms: downregulation of deoxycytidine kinase cripples drug activation, while ribonucleotide reductase overexpression replenishes nucleotide pools. Compounding these obstacles, hyperactive DNA repair machinery erects biochemical barricades against cytotoxic damage. Understanding these resistance mechanisms metabolic sabotage, genomic resilience, and epigenetic adaptations has driven the development of innovative strategies ranging from nanoparticle mediated drug delivery to synthetic lethality approaches.

Notably, the therapeutic landscape is undergoing evolving significantly through multimodal integration. Beyond conventional chemotherapy, gemcitabine now spearheads avant garde protocols combining epigenetic modulators with immune checkpoint blockade. In refractory malignancies, its role as a radiosensitizer and photodynamic therapy adjuvant has expanded the options for palliative care. Such paradigm expansion not only extends progression free survival but crucially preserves patients’ functional status,a dual triumph in oncological care.

Delving into drug resistance mechanisms unveils a complex process of clonal evolution and adaptation. Neoplastic clones exploit genomic plasticity through KRAS driven metabolic reprogramming, TGF-β-mediated immune evasion, and Notch pathway driven stemness. These molecular subterfuges necessitate a precision medicine counteroffensive: CRISPR-based functional genomics identifies vulnerability nodes, while liquid biopsy platforms track clonal evolution in real time. Therapeutically, pharmacodynamic modeling informs adaptive dosing regimens that outmaneuver resistance development, a dynamic process of adaptation and counter strategy at the molecular level.

However, the gap between clinical and translational research remains a key challenge. The predictive value of preclinical models is limited, necessitating innovative platforms to improve translational outcomes, such as clinicopathologically annotated organoid biobanks and AI driven *in silico* trials. Implementing reverse translational processes—where clinical observations directly guide basic research priorities—is crucial to overcoming this stagnation. For example, combining dynamic circulating tumor DNA monitoring with CRISPR based functional genomics could establish a virtuous cycle of discovery. Notably, phytochemicals have shown potential as novel agents to counteract resistance. Curcumin’s dual modulation of RRM2 suppression and immunogenic cell death induction, when paired with gemcitabine, achieves 40% regression in pancreatic orthografts. Curcumin reverses resistance to gemcitabine through various mechanisms such as inhibiting drug efflux pumps, regulating signaling pathways (such as PI3K/AKT), and inducing specific modes of death (such as ferroptosis) (Namwa et al., 2025; Yang et al., 2017). Similarly, astragaloside IV rewires apoptoticautophagic cross talk through non canonical NF-κB inhibition. These botanical adjuvants represent a promising approach for integrating Eastern and Western therapeutic principles.

Another critical consideration is the substantial pharmacokinetic limitations of curcumin, which are one of the core bottlenecks hindering its clinical translation. Ignoring these issues, any promising preclinical mechanism research may fail in future human trials. The DDI risk of curcumin mainly stems from its extensive inhibition of drug metabolizing enzymes and transporters. Therefore, the success of future research depends not only on in depth exploration of molecular mechanisms, but also on whether advanced drug delivery technologies (such as nano targeted drug delivery) and rational clinical design can cleverly utilize its mechanism of action while minimizing off target side effects.

Ultimately, building an effective drug resistance intelligence framework requires the integration of multiple dimensions. Single cell transcriptomics can delineate clonal architecture, organ-on-a-chip systems can accelerate drug combination testing, and blockchain-enabled data lakes can aggregate global resistance patterns. This coordinated collaboration will shift cancer management from reactive intervention to proactive control, a paradigm shift in treatment strategies.```````
